# A cross-sectional study on the concordance between vaginal HPV DNA detection and type-specific antibodies in a multi-ethnic cohort of women from Amsterdam, the Netherlands – the HELIUS study

**DOI:** 10.1186/s12879-016-1832-4

**Published:** 2016-09-22

**Authors:** Alexandra Kovaleva, Catharina J. Alberts, Tim Waterboer, Angelika Michel, Marieke B. Snijder, Wilma Vermeulen, Liza Coyer, Maria Prins, Maarten Schim van der Loeff

**Affiliations:** 1 Department of Infectious Diseases Research and Prevention, Public Health Service of Amsterdam (GGD), Nieuwe Achtergracht 100, Amsterdam, 1018 WT The Netherlands; 2 AMC Graduate School, Academic Medical Center, Meibergdreef 9, Amsterdam, 1105 AZ The Netherlands; 3 German Cancer Research Center (DKFZ), Im Neuenheimer Feld 280, Heidelberg, 69120 Germany; 4 Department of Public Health, Academic Medical Center, Meibergdreef 9, Amsterdam, 1105 AZ The Netherlands; 5 Public Health Laboratory, Public Health Service of Amsterdam (GGD), Nieuwe Achtergracht 100, Amsterdam, 1018 WT The Netherlands; 6 Department of Internal Medicine, Center for Infection and Immunity Amsterdam (CINIMA), Academic Medical Center, Meibergdreef 9, Amsterdam, 1105 AZ The Netherlands

**Keywords:** Human papillomavirus, Vaginal, Antibodies, Serology, Concordance, HELIUS study

## Abstract

**Background:**

Acquisition of genital human papillomavirus (HPV) infection is common among the young, sexually active population. Genital HPV infections do not always lead to seroconversion. We aimed to assess the association between cervico-vaginal high risk (hr) HPV DNA and type-specific antibodies in an ethnically diverse cohort of young women.

**Methods:**

Women of Dutch, South-Asian Surinamese, African Surinamese, Ghanaian, Moroccan and Turkish origin participating in a large-scale multi-ethnic population-based cohort (the HELIUS study) provided vaginal self-samples and blood samples, and completed a questionnaire regarding demographics, lifestyle and sexual health. Vaginal swabs were tested for HPV using the highly sensitive SPF10-PCR DEIA/LiPA25 system (version1). Serum samples were tested for type-specific L1 antibodies against 7 hrHPV types (16,18,31,33,45,52,58) with multiplex serology. We assessed the association between vaginal HPV DNA and type-specific seropositivity with logistic and linear regression, using generalized estimating equations (GEE). We determined whether this association varies by ethnicity by adding an interaction term.

**Results:**

We selected 532 women who completed the questionnaire, provided a vaginal swab and a blood sample. Their median age was 27 years (interquartile range 24–31 years). Prevalence of DNA of any of the 7 hrHPV was 22 %; HPV-52 was most common. Prevalence of antibodies against one or more hrHPV types was 24 %; HPV-16 seropositivity was most common. In multivariable logistic regression analysis using GEE, adjusting for other determinants, vaginal HPV DNA detection was associated with type-specific HPV seropositivity (OR 1.53, 95 % CI 1.06-2.20). In multivariable linear regression analysis using GEE, the geometric mean of type-specific antibody reactivity was 1.15 (95 % CI 1.04-1.27) times higher in women positive for HPV DNA compared to HPV DNA-negative women. There was little evidence that ethnicity modified the association between HPV DNA, and type-specific seropositivity, or with antibody reactivities (*p* = 0.47 and *p* = 0.57, respectively).

**Conclusions:**

In this multi-ethnic group of young women in Amsterdam, cervico-vaginal hrHPV DNA detection was an independent determinant of type-specific HPV seropositivity.

## Background

Due to increased globalization, European populations are becoming more ethnically diverse, and health risks and outcomes differ between ethnic groups [1]. Acquisition of human papillomavirus (HPV) infection is common among the young, sexually active population. Oncogenic human papillomaviruses cause the majority of cervical, anal, and penile cancer, and in some countries also of oropharyngeal cancer [2]. The incidence of cervical carcinoma differs between ethnic groups in the Netherlands, with higher incidence in those of the non-Dutch origin [3].

Most infections with oncogenic HPV are cleared spontaneously and pass without malignant sequelae. Estimates of proportions of women who seroconvert after infection range from 55–85 %, depending on study design and methods employed [4–6]. The median time to seroconversion after genital infection with HPV ranged from 8.3–31.3 months between studies [4–6]. In a large multi-national study 79 % of cases of cervical HPV DNA cleared within 24 months [7]. Concordance of genital HPV detection and serum antibodies is not expected per se, because the former may constitute current infection, and the latter past infection or infection of longer duration. Some studies, examining the concordance between cervico-vaginal HPV DNA and type-specific seropositivity showed positive associations, while others showed no significant relationship [8–15].

We aimed to assess the association between prevalent cervico-vaginal HPV DNA of seven high risk HPV (hrHPV) types and type-specific antibodies in serum, and to evaluate whether this association varies by ethnicity.

## Methods

### Study design

The HEalthy LIfe in an Urban Setting (HELIUS) study is a multi-ethnic cohort study conducted in Amsterdam which has been described in detail elsewhere [16]. Briefly, the study was initiated in 2011 and includes people aged 18–70 years from the 6 largest ethnic groups in Amsterdam, i.e. those of Dutch, South-Asian Surinamese, African Surinamese, Ghanaian, Moroccan and Turkish origin. Participants were randomly selected per ethnicity from the Amsterdam municipal registers. Data were collected by questionnaire and physical examination; collection of biological samples took place during physical examination. Information on demographic characteristics (age, education, marital status), health-related characteristics (parity, HPV vaccination, smoking), and sexual behavior (age at sexual debut, number of lifetime male sex partners, type of sexual partner in the preceding six months) were obtained via questionnaire.

The selection process for this study has been described in detail elsewhere (Alberts et al. 2015, manuscript submitted for review). Briefly, we selected all women aged 18–34 years from the HELIUS participants who were enrolled until December 2013, who completed the questionnaire on demographics, health behavioral characteristics, and sexual behavior, provided a self-collected vaginal swab, and of whom a blood sample was available (*n* = 959). We randomly selected a maximum of 7 women per life year per ethnic group, resulting in a selection of 610 women. Participants who were not tested for HPV antibodies (*n* = 60), who reported HPV vaccination (*n* = 10) and participants with an invalid HPV DNA test result (*n* = 8) were excluded, resulting in a study sample of 532 women. The study protocol was approved by The Academic Medical Center Ethics Review Board (Amsterdam, the Netherlands), and all participants provided written informed consent (reference number METC 10/100# 17.10.1729).

### HPV DNA detection and genotyping

Self-collected vaginal swabs (COPAN Italia, Brescia, Italy) were stored at 4 °C for a maximum of 5–6 days at the research location and sent to the Public Health Service of Amsterdam where they were stored at −20 °C until further analysis. Swabs were eluted in 600 μL PBS and DNA was extracted from 200 μL of each specimen using the isopropanol method [17] with an end volume of 100 mL. The presence of HPV in the swabs was assessed by using the highly sensitive SPF10-PCR DEIA/LiPA25 system version 1 (DDL Diagnostics Laboratory, Rijswijk, the Netherlands) [18]. Briefly, with this technique a 65 base-pair open reading frame of the L1 region was amplified with SPF10 primers resulting in biotinylated amplicons. The amplification products were tested for HPV DNA with a DNA enzyme immuno-assay by use of hybridization with a cocktail of probes that recognized at least 54 HPV types. Optical densities were measured and compared with cut-off values from internal controls. Positive samples were genotyped by a reverse hybridization line probe assay which allows simultaneous detection of HPV-6, 11, 16, 18, 31, 33–35, 39, 40, 42–45, 51–54, 56, 58, 59, 66, 68/73, 70, and 74. Prevalence of vaginal HPV DNA in this population and determinants of vaginal HPV DNA detection have been reported elsewhere (Alberts et al. 2015, manuscript submitted for review).

### HPV antibody detection

Serum samples were stored at −80 °C at the Academic Medical Center of Amsterdam until sent for analysis to the German Cancer Research Center, Heidelberg, Germany. Antibodies to the L1 proteins of HPV-16, 18, 31, 33, 45, 52 and 58 were analyzed by multiplex serology assay based on glutathione S-transferase (GST) fusion protein capture on fluorescent beads (SeroMAP Microspheres; Luminex) [19]. Antibody quantity was determined as the median R-phycoerythrin fluorescence intensity (MFI) from at least 100 beads of the same internal color. MFI values were dichotomized as antibody positive or negative based on predetermined cut-off values for each antibody by analyzing MFI values obtained from Korean HPV DNA-negative self-reported virgin females using an algorithm of the mean plus 5 SDs after exclusion of outliers [12]. This resulted in cut-offs varying from 394–712 MFI.

### Statistical analysis

Differences in baseline characteristics between ethnic groups were assessed using the Pearson’s chi-squared or the Kruskal-Wallis test as appropriate. The statistical analysis was restricted to HPV types 16, 18, 31, 33, 45, 52 and 58.

First, we assessed type-specific HPV seropositivity on a dichotomous scale among both HPV DNA positive women and HPV DNA negative women per individual HPV type by performing standard univariable logistic analyses. As a woman can be infected with multiple HPV types simultaneously we also analyzed data on all 7 HPV types with univariable and multivariable logistic regression models using generalized estimating equations (GEE). We used a binomial distribution and an exchangeable correlation structure with robust variance.

We then investigated the association between cervico-vaginal HPV DNA and type-specific antibody reactivities using linear regression. For this purpose, we transformed antibody reactivities to their natural logarithm. We first undertook standard univariable and multivariable linear regression for individual HPV types. Next, we proceeded with univariable and multivariable linear regression using GEE. Because the mean and standard deviation of antibody reactivities of the 7 hrHPV types differed, we standardized the natural logarithm of antibody reactivity levels by calculating Z-scores. We used a Gaussian distribution and an exchangeable correlation structure, with robust variance. Because we included a log-transformed outcome variable in the linear regression analysis, we back-transformed the regression coefficients in order to ease interpretation. As a result, the coefficients can be interpreted as a multiplicative factor of the geometric mean; in other words the geometric mean of an antibody reactivity in women who are positive for cervico-vaginal HPV DNA, is equal to that factor times the geometric mean of women who are HPV DNA negative.

All multivariable models were adjusted for an a priori defined set of variables, i.e. age, ethnicity, marital status, smoking in pack years, and number of lifetime male sexual partners. We included age as a continuous variable using restricted cubic splines with 4 knots. All variables other than age were included as categorical variables. No backward selection was done.

Only one sexual risk variable could be included in the model because of multicollinearity between these variables. Also, only smoking in pack years and not smoking status was included in the models because of multicollinearity. For all other variables multicollinearity did not appear to be a concern.

As a last step we investigated whether the association between HPV seropositivity and HPV DNA varied by ethnicity. To do this we tested for interaction by adding an interaction term to the multivariable model. Stata Statistical Software release 13.1 (StataCorp, College Station, TX, USA) was used for statistical analyses.

## Results

A total of 532 women were included in the analyses (Table 1). The average age of the participants did not differ between ethnic groups (median 27 years, interquartile range (IQR) 23–30 years). All other baseline characteristics differed across ethnic groups; we found significant differences in education, marital status, and smoking. Furthermore, lifetime male sexual partners, age of sexual debut and type of sexual contact in in the last six months varied significantly by ethnicity. Most women reported having one steady sexual partner in the preceding six months.

**Table 1:** Demographic, health and sexual behavior characteristics by ethnicity of 532 women from Amsterdam, the Netherlands

	Dutch		South-Asian Surinamese		African Surinamese		Ghanaian		Moroccan		Turkish			Total	
	*N* = 98		*N* = 88		*N* = 100		*N* = 68		*N* = 101		*N* = 77			*N* = 532	
	n	%	n	%	n	%	n	%	n	%	n	%	*P*-value	n	%
Age in years															
Median (IQR)	27 (23–31)	27 (23–31)	26 (22–30)	26 (21–30)	27 (23–30)	27 (23–31)	0.46	27 (23–30)							
18–22	23	23 %	19	22 %	27	27 %	22	32 %	20	20 %	16	21 %	0.98	127	24 %
23–26	24	24 %	21	24 %	25	25 %	16	24 %	28	28 %	17	22 %		131	25 %
27–30	26	27 %	26	30 %	25	25 %	15	22 %	28	28 %	24	31 %		144	27 %
31–34	25	26 %	22	25 %	23	23 %	15	22 %	25	25 %	20	26 %		130	24 %
Education^a^															
Low/Intermediate	28	29 %	56	64 %	67	68 %	50	75 %	66	65 %	58	75 %	<0.001	325	61 %
High	70	71 %	32	36 %	32	32 %	17	25 %	35	35 %	19	25 %		205	39 %
Marital status															
Married/Cohabitating	30	31 %	29	33 %	13	13 %	14	21 %	39	39 %	36	47 %	<0.001	161	30 %
Never married	68	69 %	54	61 %	86	86 %	53	78 %	52	51 %	35	45 %		348	65 %
Divorced	0	0 %	5	6 %	1	1 %	1	1 %	10	10 %	6	8 %		23	4 %
Smoking status															
Never	47	48 %	61	69 %	63	64 %	66	97 %	81	80 %	36	47 %	<0.001	354	67 %
Former	20	20 %	8	9 %	11	11 %	2	3 %	6	6 %	10	13 %		57	11 %
Current	31	32 %	19	22 %	25	25 %	0	0 %	14	14 %	31	40 %		120	23 %
Smoking in pack years															
Never	47	48 %	61	69 %	63	64 %	66	97 %	81	80 %	36	47 %	<0.001	354	67 %
< 2 pack years	24	25 %	14	16 %	22	22 %	1	1 %	9	9 %	13	17 %		83	16 %
> 2 pack years	26	27 %	13	15 %	14	14 %	1	1 %	11	11 %	27	36 %		92	17 %
Age of sexual debut in years															
Median (IQR)^b^	16 (15–18)	18 (17–20)	17 (15–18)	18 (16–19)	19 (17–22)	20 (18–22)	<0.001	18 (16–20)							
Never had sex	5	5 %	14	16 %	8	8 %	18	28 %	33	33 %	15	20 %	<0.001	93	18 %
≥ 21	9	9 %	12	14 %	3	3 %	9	14 %	29	29 %	26	34 %		88	17 %
19-20	8	8 %	16	19 %	17	17 %	5	8 %	12	12 %	16	21 %		74	14 %
18-17	27	28 %	29	34 %	27	27 %	19	30 %	13	13 %	14	18 %		129	25 %
≤ 16	49	50 %	15	17 %	45	45 %	13	20 %	13	13 %	5	7 %		140	27 %
Number of lifetime male sexual partners															
Median (IQR)	6 (3–11)	2 (1–4)	5 (2–8)	2 (0–4)	1 (0–1)	1 (1–2)	<0.001	3 (1–7)							
None^c^	5	5 %	14	16 %	9	9 %	18	28 %	34	34 %	16	21 %	<0.001	96	18 %
1	8	8 %	27	31 %	6	6 %	11	17 %	44	44 %	38	50 %		134	26 %
2–5	33	34 %	34	40 %	42	42 %	25	39 %	15	15 %	12	16 %		161	31 %
6–10	25	26 %	9	10 %	26	26 %	8	13 %	4	4 %	9	12 %		81	15 %
≥ 11	27	28 %	2	2 %	16	16 %	2	3 %	3	3 %	1	1 %		51	10 %
Type of sexual contact past 6 months															
Never had sex	5	5 %	14	16 %	8	8 %	18	28 %	33	33 %	15	20 %	<0.001	93	18 %
No sex	17	17 %	14	16 %	11	11 %	9	14 %	16	16 %	11	14 %		78	15 %
With steady partner only	55	56 %	55	64 %	60	60 %	34	53 %	49	49 %	39	51 %		292	56 %
With casual partner^d^	21	21 %	3	3 %	21	21 %	3	5 %	2	2 %	11	14 %		61	12 %

IQR, interquartile range

*P*-values of categorical variables are based on Chi-squared test and p of continuous variables are based on the Kruskal-Wallis test

Missing values for: education, *n* = 2; age sexual debut, *n* = 8; lifetime male sexual partners, *n* = 9; type of sexual partner past 6 months, *n* = 8; smoking status, *n* = 1; smoking in pack years, *n* = 3. ^a^ Education was categorized as low or intermediate education if participant had (1) never been to school or had primary schooling only, (2) followed vocational schooling or lower secondary schooling, or (3) followed intermediate/higher secondary education schooling; and was categorized as high education if participant had followed higher vocational schooling or university. ^b^Of those women who reported ever having sex. ^c^This category includes women who never had sex and those who exclusively had sexual contact with women, therefore this category has a different number than the category “Never had sex” in the variables “Age of sexual debut in years” and “Type of sexual contact past 6 months”. ^d^And with or without steady partner

Vaginal HPV DNA of at least one of the 7 hrHPV types was detected in 115 (22 %) women of whom some had multiple infections with different HPV types; DNA of HPV-52 was detected most often (Table 2). Of the 532 women, 130 (24 %) were seropositive for at least one of the 7 hrHPV types; HPV-16 had the highest seroprevalence (14 %). Among women positive for DNA of an individual HPV type, seropositivity for the corresponding HPV type ranged from 13–33 %. Among HPV DNA negative women, seropositivity ranged from 6–13 %.

**Table 2:** Vaginal hrHPV DNA and type-specific seropositivity^a^, and association of vaginal hrHPV DNA with type-specific antibodies^b^

HPV type^c^	HPV DNA detected n (%)	HPV seropositive n (%)	Seropositive among DNA + n (%)^d^	Seropositive among DNA - n (%)^e^	Crude OR for seropositivity (95 % CI)^f^
16	32 (6 %)	73 (14 %)	6 (19 %)	67 (13 %)	1.49 (0.59,3.76)
18	20 (4 %)	59 (11 %)	4 (20 %)	55 (11 %)	2.08 (0.67,6.44)
31	28 (5 %)	68 (13 %)	6 (21 %)	62 (12 %)	1.94 (0.76,4.98)
33	7 (1 %)	36 (7 %)	2 (29 %)	34 (6 %)	5.78 (1.08,30.88)
45	8 (2 %)	51 (10 %)	1 (13 %)	50 (10 %)	1.35 (0.16,11.23)
52	43 (8 %)	54 (10 %)	9 (21 %)	45 (9 %)	2.61 (1.18,5.97)
58	9 (2 %)	46 (9 %)	3 (33 %)	43 (8 %)	5.58 (1.35,23.10)

CI, confidence interval; hrHPV, high risk human papillomavirus; IQR, interquartile range; OR, odds ratio. ^a^ Among 532 women from a population-based cohort (HELIUS), Amsterdam, the Netherlands. ^b^Univariable analysis conducted with logistic regression. ^c^ Cut-offs used in this study: 422 MFI for HPV-16, 394 for HPV-18, 712 for HPV-31, 515 for HPV-33, 368 for HPV-45, 547 for HPV-52, and 371 MFI for HPV-58. ^d^Among number of HPV DNA positive women in column 2. ^e^Among number of HPV DNA negative women, i.e. the number cited in column 2 subtracted from 532. ^f^Multivariable analysis was not undertaken because of small numbers

For all 7 HPV types we found that HPV DNA positive women were seropositive for the corresponding type more often than HPV DNA negative women (indicated by ORs > 1 for all), but this was only significant for HPV-33, 52 and 58 (Table 2). In univariable logistic regression analysis using GEE cervico-vaginal HPV DNA detection was significantly associated with type-specific seropositivity (OR 1.70, 95 % CI 1.22 – 2.36) (Table 3). After adjustment for age, ethnicity, marital status, smoking in pack years, and number of lifetime male sexual partners the association remained positive and statistically significant (OR 1.53, 95 % CI 1.06 – 2.20).

**Table 3:** Associations of risk factors and seropositivity for seven hrHPV types^a^; logistic regression using GEE

	Crude OR	95 % CI	*P*-value	Adjusted OR^b^	95 % CI	*P*-value
Cervico-vaginal HPV DNA						
No	1		0.002	1		0.02
Yes	1.70	(1.22,2.36)		1.53	(1.06,2.20)	
Age in years^c^						
19	2.76	(1.34,5.70)		4.33	(2.07,9.06)	
24	1		0.02	1		<0.001
29	1.07	(0.58,1.99)		1.10	(0.60,2.05)	
34	1.53	(0.73,3.20)		1.53	(0.72,3.24)	
Ethnicity^d^						
Dutch	1		<0.001	1		0.22
South-Asian Surinamese	0.63	(0.30,1.31)		1.39	(0.65,2.96)	
African Surinamese	1.80	(0.99,3.26)		1.96	(1.08,3.55)	
Ghanaian	0.74	(0.34,1.59)		1.11	(0.48,2.55)	
Moroccan	0.43	(0.20,0.94)		1.30	(0.57,2.96)	
Turkish	0.36	(0.14,0.88)		0.85	(0.34,2.15)	
Marital status^e^						
Ever married/Cohabitating	1		0.002	1		0.06
Never married	2.22	(1.33,3.69)		1.72	(0.98,3.03)	
Smoking status						
Never smoked	1		0.50			
Former smoker	1.42	(0.74,2.73)				
Current smoker	1.22	(0.73,2.03)				
Smoking in pack years						
Never smoked	1		0.023	1		0.04
< 2 years	1.92	(1.14,3.22)		1.45	(0.84,2.50)	
≥ 2 years	0.81	(0.43,1.52)		0.57	(0.30,1.09)	
Age of sexual debut						
Never had sex	1		<0.001			
≥ 21	1.31	(0.44,3.89)				
19-20	2.29	(0.82,6.38)				
18-17	4.03	(1.65,9.85)				
≤ 16	5.50	(2.30,13.17)				
Lifetime male sexual partners						
None	1		<0.001	1		<0.001
1	1.88	(0.73,4.85)		3.61	(1.37,9.49)	
2–5	3.44	(1.43,8.27)		4.29	(1.72,10.74)	
6–10	6.27	(2.54,15.49)		11.2	(4.13,30.33)	
≥ 11	7.08	(2.74,18.32)		12.07	(4.09,35.57)	

CI, confidence interval; GEE, generalized estimating equations; hrHPV, high risk human papillomavirus; OR, odds ratio. ^a^Among 532 women from the population-based cohort HELIUS, Amsterdam, the Netherlands, 2011–2013. ^b^Multivariable models adjusted for age in years, ethnicity, marital status, smoking in pack years, number of lifetime male sexual partners. ^c^For analytic purposes age was modeled using restricted cubic splines with knots at the 5th, 35th, 65th and 95th percentile. The ORs of four ages from the total analysis with five years in between have been chosen for display in the table. ^d^Effect estimates per ethnicity of vaginal hrHPV DNA detection for seropositivity in ORs (95 % CI): Dutch 1.74 (0.99,3.09), South-Asian Surinamese 2.13 (0.72,6.33), African Surinamese 1.43 (0.82,2.45), Ghanaian 0.93 (0.25,3.48), Moroccan 0.67 (0.13,3.51), Turkish 4.01 (1.44,11.12). ^e^For marital status, the small category of divorced women was added to the category of married or cohabitating women to prevent instability of the model

The geometric mean of the antibody reactivities in MFI was higher among women who were positive for cervico-vaginal HPV DNA. This was the case for all 7 hrHPV types, though only significant for HPV-31, 52 and 58 (Table 4). When we adjusted for age, ethnicity, marital status, smoking in pack years, and lifetime number of male sexual partners in multivariable linear regression models, DNA detection of HPV-31 remained significantly associated with a higher antibody reactivity for this type. For all other HPV types the back-transformed regression coefficient was >1, but the association was not significant.

**Table 4:** Linear regression of antibody reactivities (MFI) and type-specific vaginal HPV detection; univariable and multivariable analyses

			Univariable			Multivariable^a^		
Vaginal HPV type detection	Geometric mean of antibody reactivities in MFI among DNA negative (95 % CI)	Geometric mean of antibody reactivities in MFI among DNA positive (95 % CI)	Back-transformed regression coefficient^b^	95 % CI	*P*-value	Back-transformed regression coefficient^b^	95 % CI	*P*-value
HPV-16	80 (70–90)	110 (72–167)	1.39	(0.83,2.31)	0.21	1.34	(0.82,2.17)	0.24
HPV-18	104 (95–112)	134 (83–216)	1.30	(0.84,2.00)	0.24	1.26	(0.81,1.93)	0.30
HPV-31	141 (126–157)	303 (203–455)	2.16	(1.34,3.48)	<0.001	1.68	(1.03,2.72)	0.04
HPV-33	80 (73–88)	128 (40–411)	1.59	(0.70,3.61)	0.26	1.17	(0.53,2.57)	0.69
HPV-45	78 (71–85)	120 (51–280)	1.54	(0.73,3.23)	0.26	1.34	(0.66,2.70)	0.42
HPV-52	90 (81–100)	147 (99–218)	1.63	(1.13,2.36)	0.01	1.30	(0.91,1.87)	0.15
HPV-58	85 (79–92)	165 (72–376)	1.93	(1.05,3.55)	0.03	1.52	(0.84,2.75)	0.16

CI, confidence interval; MFI, median fluorescence intensity. ^a^Multivariable models adjusted for age in years, ethnicity, marital status, smoking in pack years, number of lifetime male sexual partners. For analytic purposes age was modeled using restricted cubic splines with knots at the 5th, 35th, 65th and 95th percentile. ^b^The back-transformed regression coefficient for antibody reactivities is interpreted as being the factor by which the geometric mean of antibody reactivities of vaginal HPV DNA positive women is higher in comparison to women who do not have vaginal HPV DNA. For example, women in whom vaginal HPV-31 DNA was detected have a median antibody reactivity which is 2.16 times higher than the antibody reactivity of HPV-31 DNA negative women

Next, we investigated the association between cervico-vaginal HPV DNA and standardized antibody reactivities with linear regression analysis using GEE. In univariable linear analysis with GEE women with prevalent cervico-vaginal HPV DNA had higher type-specific antibody reactivities than women without cervico-vaginal HPV DNA (back-transformed regression coefficient 1.15 [95 % CI 1.04 – 1.27]) (Table 5). In the multivariable analysis the back-transformed regression coefficient remained unchanged (1.15 [95 % CI 1.04 – 1.27]). This indicates that women with cervico-vaginal HPV DNA have higher type-specific antibody reactivities than women without cervico-vaginal HPV DNA, independent of other determinants.

**Table 5:** Associations of antibody reactivities for seven hrHPV^a^; linear regression using GEE^b^

	Back-transformed regression coefficient^c^	95 % CI	*P*-value	Adjusted back-transformed regression coefficient^c^	95 % CI	*P*-value
Cervico-vaginal HPV DNA						
No	1		0.005	1		0.007
Yes	1.15	(1.04,1.27)		1.15	(1.04,1.27)	
Age in years^d^						
19	1.47	(1.11,1.95)		1.54	(1.18,2.01)	
24	1		0.58	1		0.013
29	1.17	(0.95,1.43)		1.10	(0.90,1.34)	
34	1.15	(0.89,1.48)		1.08	(0.84,1.39)	
Ethnicity^e^						
Dutch	1		<0.001	1		<0.001
South-Asian Surinamese	1.09	(0.86,1.39)		1.45	(1.13,1.85)	
African Surinamese	1.57	(1.25,1.98)		1.62	(1.29,2.03)	
Ghanaian	1.14	(0.89,1.48)		1.39	(1.07,1.82)	
Moroccan	0.76	(0.60,0.96)		1.13	(0.88,1.45)	
Turkish	0.73	(0.57,0.93)		1.07	(0.82,1.40)	
Marital status^f^						
Ever married/Cohabitating	1		<0.001	1		0.05
Never married	1.30	(1.11,1.51)		1.19	(1.00,1.43)	
Smoking status						
Never smoked	1		0.73			
Former smoker	1.10	(0.86,1.40)				
Current smoker	0.99	(0.83,1.19)				
Smoking in pack years						
Never smoked	1		0.13	1		0.09
< 2 years	1.18	(0.96,1.45)		1.01	(0.82,1.24)	
≥ 2 years	0.91	(0.74,1.11)		0.81	(0.66,0.99)	
Age of sexual debut in years						
Never had sex	1		<0.001			
≥ 21	1.15	(0.90,1.47)				
19-20	1.36	(1.06,1.76)				
18-17	1.65	(1.32,2.07)				
≤ 16	1.94	(1.56,2.41)				
Lifetime male sexual partners						
None	1		<0.001	1		<0.001
1	1.23	(0.99,1.53)		1.52	(1.20,1.92)	
2–5	1.60	(1.30,1.98)		1.66	(1.32,2.09)	
6–10	1.88	(1.47,2.40)		2.14	(1.63,2.82)	
≥ 11	2.35	(1.77,3.11)		2.85	(2.06,3.95)	

CI, confidence interval; GEE, generalized estimating equations; hrHPV, high risk human papillomavirus. ^a^ Among 532 women from the population-based cohort HELIUS, Amsterdam, the Netherlands, 2011–2013. ^b^For this analysis the antibody reactivity was transformed to its natural logarithm, and subsequently standardized by calculating Z-scores. ^c^The back-transformed regression coefficient is interpreted as being the factor by which the standardized antibody reactivities of women are higher in comparison to women in the reference category, which in this analysis is the HPV DNA negative group. ^d^ For analytic purposes age was modeled using restricted cubic splines with knots at the 5th, 35th, 65th and 95th percentile. The back-transformed regression coefficients of four ages from the total analysis with five years in between have been chosen for display in the table. ^e^ Effect estimates per ethnicity of vaginal hrHPV DNA detection for antibody reactivity in back-transformed regression coefficient (95 % CI): Dutch 1.19 (0.97,1.45), South-Asian Surinamese 1.21 (0.91,1.62), African Surinamese 0.98 (0.81,1.19), Ghanaian 0.99 (0.73,1.36), Moroccan 1.29 (1.00,1.66), Turkish 1.26 (0.98,1.61). ^f^For marital status, the small category of divorced women was added to the category of married or cohabitating women to prevent instability of the model. Multivariable models adjusted for age in years, ethnicity, marital status, smoking in pack years, number of lifetime male sexual partners

We examined whether the effect of HPV DNA on seropositivity differed by ethnicity by adding an interaction term to the models. Neither in logistic, nor in linear regression analyses using GEE did we detect significant effect modification (*p* = 0.47 and *p* = 0.57, respectively).

## Discussion

Our results show that in this multi-ethnic group of young women in Amsterdam, cervico-vaginal HPV DNA detection is an independent risk factor of type-specific HPV seropositivity and that detection of cervico-vaginal HPV DNA is associated with higher antibody reactivities of type-specific antibodies.

Variables that describe sexual behavior directly or that could be considered a proxy for sexual behavior were strongly associated with higher type-specific HPV antibody reactivities and HPV seropositivity. The latter has also been shown by several other studies [8, 20, 21].

Since antibody formation in response to natural infection is a slow process that could extend to over a year, we expected in this young study population that concordance between cervico-vaginal DNA and antibodies would be low. Our study population however had a significant positive association even after adjustment for other well-known risk factors. Our study population most likely represents women who contracted their HPV infection relatively recently. A small proportion of these infections will become persistent and the majority will be cleared [22, 23].

With these results we aim to show that the interpretation of HPV (sero-)prevalences may not be as straightforward as sometimes is depicted, i.e. even though there is a significant positive correlation between HPV DNA and HPV seropositive participants, being HPV DNA positive does not imply that a person inevitably will be or will become HPV seropositive, while being HPV seronegative does not necessarily mean an individual has never been exposed. Concordance between HPV DNA and seropositivity therefore has little clinical application as a diagnostic tool. However it may be valuable in describing the natural history of HPV infection.

Because seropositivity to HPV is a measure of lifetime exposure to HPV we expected that age would be associated with HPV seropositivity. In our sample we found a significant positive association with age 19 and seropositivity relative to the reference age of 24 years, whereas the odds for seropositivity seem to not vary much with 29 and 34 years. A similar trend has been reported by Scherpenisse et al. (2012); seropositivity percentages rise sharply in the early twenties, then stabilize and do not vary much until 59 years of age [24].

Both significant and non-significant associations between cervical HPV DNA and type-specific seropositivity have previously been reported [8–15]. We found a significant positive association in our sample between cervico-vaginal HPV DNA and type-specific seropositivity expressed on a dichotomous scale, and further strengthened this conclusion by confirming it in an analysis with the outcome measure on a continuous scale. Our finding of a positive association between cervico-vaginal HPV DNA and type-specific antibodies is in agreement with several previous studies [9, 12–15]. We complement and expand available knowledge on HPV by including seven hrHPV types in our analysis, where most previous studies included fewer types.

Some previous studies differ in methodology to ours in several respects, in that they were conducted amongst women at higher risk for HPV infections (more sexual risk behavior or patients with cervical dysplasia or recruitment in STI clinics) [9, 13, 14]. In other publications, type-specific positive associations have also been found in young women that were recruited from populations not at high risk of HPV infection [12, 15].

The fact that we could only include women who provided a cervico-vaginal swab might have introduced some selection bias into our study. Alberts et al. (2015) have shown that women of Turkish and Moroccan origin from the same study population who declined to provide a sample exhibited less sexual risk behavior (Alberts et al., 2015, submitted for review). However, no significant differences were observed between women who did and did not provide a self-swab for the other ethnicities suggesting limited bias for these groups. The ethnic diversity of our sample, and the concurrent analysis of 7 hrHPV types are strengths of this study. Because seropositivity is a measure of lifetime exposure to HPV and not necessarily the result of a current infection, an association between cervico-vaginal HPV DNA and seropositivity can be difficult to interpret.

## Conclusions

In conclusion, we observed a positive association between cervico-vaginal DNA detection and type-specific seropositivity. Moreover, we demonstrated higher type-specific antibody reactivities in women who are HPV DNA positive compared to HPV DNA negative women in a multi-ethnic urban population of young women. These associations were independent of demographic and behavioral factors. There was little evidence that ethnicity constituted a significant effect modifier in our population which suggests that effect modifiers other than genetic variation are at play that result in differing HPV infection outcomes in ethnic minorities.

## Abbreviations

CI: confidence interval
GEE: generalized estimating equations
HELIUS: HEalthy LIfe in an Urban Setting
HPV: human papillomavirus
hrHPV: high-risk human papillomavirus
IQR: interquartile range
MFI: median fluorescence intensity
OR: odds ratio

## References

[1] Keppel KG, Pearcy JN, Heron MP (2010). Is there progress toward eliminating racial/ethnic disparities in the leading causes of death?. Public Health Rep.

[2] de Martel C, Ferlay J, Franceschi S, Vignat J, Bray F, Forman D, Plummer M (2012). Global burden of cancers attributable to infections in 2008: a review and synthetic analysis. Lancet Oncol.

[3] Arnold M, Aarts MJ, van der Aa M, Visser O, Coebergh JW (2013). Investigating cervical, oesophageal and colon cancer risk and survival among migrants in The Netherlands. Eur J Public Health.

[4] Carter JJ, Koutsky LA, Hughes JP, Lee SK, Kuypers J, Kiviat N, Galloway DA (2000). Comparison of human papillomavirus types 16, 18, and 6 capsid antibody responses following incident infection. J Infect Dis.

[5] Ho GY, Studentsov YY, Bierman R, Burk RD (2004). Natural history of human papillomavirus type 16 virus-like particle antibodies in young women. Cancer Epidemiol Biomarkers Prev.

[6] Steele J, Collins S, Wen K, Ryan G, Constandinou-Williams C, Woodman CB (2008). Measurement of the humoral immune response following an incident human papillomavirus type 16 or 18 infection in young women by a pseudovirion-based neutralizing antibody assay. Clin Vaccine Immunol.

[7] Jaisamrarn U, Castellsague X, Garland SM, Naud P, Palmroth J, Del Rosario-Raymundo MR, Wheeler CM, Salmeron J, Chow SN, Apter D (2013). Natural history of progression of HPV infection to cervical lesion or clearance: analysis of the control arm of the large, randomised PATRICIA study. PloS One.

[8] Viscidi RP, Ahdieh-Grant L, Clayman B, Fox K, Massad LS, Cu-Uvin S, Shah KV, Anastos KM, Squires KE, Duerr A (2003). Serum immunoglobulin G response to human papillomavirus type 16 virus-like particles in human immunodeficiency virus (HIV)-positive and risk-matched HIV-negative women. J Infect Dis.

[9] Vriend HJ, Bogaards JA, van der Klis FR, Scherpenisse M, Boot HJ, King AJ, van der Sande MA, Medical Microbiological Laboratories MHS (2013). Patterns of human papillomavirus DNA and antibody positivity in young males and females, suggesting a site-specific natural course of infection. PloS One.

[10] Paaso AE, Louvanto K, Syrjanen KJ, Waterboer T, Grenman SE, Pawlita M, Syrjanen SM (2011). Lack of type-specific concordance between human papillomavirus (HPV) serology and HPV DNA detection in the uterine cervix and oral mucosa. J Gen Virol.

[11] Castro FA, Dominguez A, Puschel K, Van De Wyngard V, Snijders PJ, Franceschi S, Pawlita M, Ferreccio C (2014). Serological prevalence and persistence of high-risk human papillomavirus infection among women in Santiago, Chile. BMC Infect Dis.

[12] Clifford GM, Shin HR, Oh JK, Waterboer T, Ju YH, Vaccarella S, Quint W, Pawlita M, Franceschi S (2007). Serologic response to oncogenic human papillomavirus types in male and female university students in Busan, South Korea. Cancer Epidemiol Biomarkers Prev.

[13] Bontkes HJ, de Gruijl TD, Walboomers JM, Schiller JT, Dillner J, Helmerhorst TJ, Verheijen RH, Scheper RJ, Meijer CJ (1999). Immune responses against human papillomavirus (HPV) type 16 virus-like particles in a cohort study of women with cervical intraepithelial neoplasia. II. Systemic but not local IgA responses correlate with clearance of HPV-16. J Gen Virol.

[14] Wu X, Zhang C, Feng S, Liu C, Li Y, Yang Y, Gao J, Li H, Meng S, Li L (2009). Detection of HPV types and neutralizing antibodies in Gansu province, China. J Med Virol.

[15] Skjeldestad FE, Mehta V, Sings HL, Ovreness T, Turpin J, Su L, Boerckel P, Roberts C, Bryan J, Jansen KU (2008). Seroprevalence and genital DNA prevalence of HPV types 6, 11, 16 and 18 in a cohort of young Norwegian women: study design and cohort characteristics. Acta Obstet Gynecol Scand.

[16] Stronks K, Snijder MB, Peters RJ, Prins M, Schene AH, Zwinderman AH (2013). Unravelling the impact of ethnicity on health in Europe: the HELIUS study. BMC Public Health.

[17] van Houdt R, Bruisten SM, Geskus RB, Bakker M, Wolthers KC, Prins M, Coutinho RA (2010). Ongoing transmission of a single hepatitis B virus strain among men having sex with men in Amsterdam. J Viral Hepat.

[18] Molijn A, Kleter B, Quint W, van Doorn LJ (2005). Molecular diagnosis of human papillomavirus (HPV) infections. J Clin Virol.

[19] Waterboer T, Sehr P, Michael KM, Franceschi S, Nieland JD, Joos TO, Templin MF, Pawlita M (2005). Multiplex human papillomavirus serology based on in situ-purified glutathione s-transferase fusion proteins. Clin Chem.

[20] Markowitz LE, Sternberg M, Dunne EF, McQuillan G, Unger ER (2009). Seroprevalence of human papillomavirus types 6, 11, 16, and 18 in the United States: National Health and Nutrition Examination Survey 2003–2004. J Infect Dis.

[21] Nielsen A, Kjaer SK, Munk C, Iftner T (2008). Type-specific HPV infection and multiple HPV types: prevalence and risk factor profile in nearly 12,000 younger and older Danish women. Sex Transm Dis.

[22] Hildesheim A, Schiffman MH, Gravitt PE, Glass AG, Greer CE, Zhang T, Scott DR, Rush BB, Lawler P, Sherman ME (1994). Persistence of type-specific human papillomavirus infection among cytologically normal women. J Infect Dis.

[23] Castle PE, Schiffman M, Herrero R, Hildesheim A, Rodriguez AC, Bratti MC, Sherman ME, Wacholder S, Tarone R, Burk RD (2005). A prospective study of age trends in cervical human papillomavirus acquisition and persistence in Guanacaste, Costa Rica. J Infect Dis.

[24] Scherpenisse M, Mollers M, Schepp RM, Boot HJ, Meijer CJ, Berbers GA, van der Klis FR, de Melker HE (2012). Changes in antibody seroprevalence of seven high-risk HPV types between nationwide surveillance studies from 1995–96 and 2006–07 in The Netherlands. PloS One.

